# Study of burnout syndrome, job satisfaction and related factors among health care workers in rural areas of Southeastern Iran

**DOI:** 10.3934/publichealth.2020014

**Published:** 2020-03-19

**Authors:** Roghayeh Ershad Sarabi, Rezvan Javanmard, Parvin Mangolian Shahrbabaki

**Affiliations:** 1 Assistant Professor, Management and Leadership in Medical Education Research Center, Kerman University of Medical Sciences, Kerman, Iran; 2 MSc in Community Health Education, Iranshahr University of Medical Sciences, Iranshahr, Iran; 3 Assistant Professor, Nursing Research Center, Razi Faculty of Nursing and Midwifery, Kerman University of Medical Sciences, Kerman, Iran

**Keywords:** burnout syndrome, job satisfaction, health care workers, health, rural areas

## Abstract

**Background:**

Burnout syndrome is a Psycho-somatic state resulting from prolonged exposure to job stressors that leads to negative self-concept, Job dissatisfaction and lack of communication with the client. Rural health centers because of lack of facilities and lack of knowledge of the service users are characterized by a high level of work-related stress, a factor known to increase the risk of burnout syndrome. The purpose of this study was to determine burnout syndrome and Job Satisfaction among health care workers in rural areas of southeastern Iran.

**Methods:**

This is a cross-sectional study that has been conducted among 225 of the healthcare workers with more than five years of experience using simple random sampling method. Data were collected by a Maslach's burnout inventory and Smith's job satisfaction questionnaire. Data were analyzed using dependent and independent t-test, one-way ANOVA and Pearson correlation coefficient.

**Results:**

The results of this study showed that the rate of burnout and job satisfaction score in health centers in rural areas was moderate. In job satisfaction, the highest score was related to the colleague and the lowest score was related to salaries and benefits. The emotional exhaustion had a negative significant effect on job satisfaction (p < 0.01).

**Conclusion:**

Considering the importance of rural community health, burnout status of health care workers should be improved. It is necessary to provide appropriate interventions to decrease stress relating to role conflict, professional communications, factors related to the families and work load.

## 1. Introduction

The most valuable asset of every organization is its human resource. An important issue to consider within any organization is its employees' job satisfaction [1]. Meanwhile, healthcare and medical centers have a special role in the community due to the significance of their role in the prevention, care, and treatment of the disease [2]. Since the efficiency of the organizations is significant, therefore, paying due attention to its human resources' motivation and affects gains the highest priority. Thus, investigations and recommendation of solutions for enhancing job satisfaction and at the same time organizational efficiency are considered to be really significant [3]. While attempting to adjust with social and occupational environment, there may be some limitations and pressures put on the individual affecting his satisfaction and will result in incompatibilities and therefore the individual wouldn't be able to act as usual and will be afflicted with job burnout.

The permanent feeling of pressure will harm individual and it's called job burnout [4]. The first theoretical explanation of job burnout has been provided by Maslach. According to this theory, the three dimensions of job burnout including emotional exhaustion, personality transformation, and lack of individual success have been explained [5]. Those with job burnout often complain about headache, sleep disorders, irritable mood, problems in marital life, anxiety, depression, high blood pressure, among others [6]. Among the most significant organizational impacts, we can refer to Being absent from work, job quit, successive delays and reduction of an individual's performance quality, various psychological complaints, conflicts with colleagues, low spirit and Job satisfaction [7].

Job satisfaction is defined as the extent to which employees feels self-motivated and satisfied with their job. Job satisfaction happens when employees feel they having career growth, and job balance [8]. Job satisfaction has been linked to important variables, including productivity, absenteeism, turnover, etc. It is significant because a person's beliefs may affect their behaviors and higher productivity [9].

According to the field studies, healthcare sector workers experience a higher rate of job burnout and it has turned to a prevalent problem in all healthcare systems worldwide [10]. It seems that the healthcare worker is one of those jobs in which the employees spend many hours providing services to their receivers. This will result in emptying their emotional reserves and this is the most fundamental symptom of job burnout [11].

Local public healthcare centers are the most local centers for providing healthcare services in the national healthcare system [12]. In Iran local healthcare center may cover one too many villages depending on geographical conditions, especially the roads and population. The only kind of personnel employed in these local healthcare centers are male/female healthcare workers. Most of the services provided by these healthcare workers are not considered as common services provided by an employee and the provided services mainly are the daily contacts with rural area's inhabitants and families. The realization of the objectives considered for these services depends on their continuity, spending longer times and having higher patience. Therefore, these common criteria are not measurable. The compulsory residence of the healthcare workers in this job during their whole occupation and lack of possibility for scientific and occupational promotion as well as the necessity of inhabiting the village during their working period are considered as two main problems for healthcare workers [13].

Considering the increasing development and application of healthcare services in different medical and hygiene fields on the one hand and scarcity of the related studies in Iran on the other hand and considering the distribution of the population and long-distance and unfavorable climatic conditions in southeastern Iran city, especially its rural areas and the shortage of welfare services and lack of the research undertaken on job burnout and its correlation with healthcare workers in in rural areas, the objective of this study has been to determine burnout syndrome and job satisfaction among health care workers in rural areas of southeastern Iran.

## 2. Method and materials

### 2.1. Study design

This is a descriptive-analytical cross-sectional study to determine burnout syndrome and Job Satisfaction among health care workers in rural areas of southeastern Iran. This study lasted from July 2018 to October 2018.

### 2.2. Instrument

Three questionnaires were used to collect data. The first part included demographic information and was researcher-made; the second part included first edition of Maslach's job burnout questionnaire [5]; and the third part included job description questionnaire [14].

Demographic information including age, years of experience, sex, level of education, marital status, place of residence, years of residence in the location, years of residence in the location, residence status, number of affiliate villages and number of children.

Maslach's questionnaire included 22 items and it measured three dimensions of job burnout such as emotional exhaustion, personality transformation and reduced individual success. All 22 items are measured through a likert scale from zero to six (never = zero and always = six). The higher emotional exhaustion and personality transformation score and lower individual success score illustrates higher rate of job burnout. The obtained score in the general aspect of job burnout and all its dimensions has been measured based on the Maslach Burnout Inventory. Internal consistency was estimated by Cronbach's coefficient alpha, which yielded reliability coefficients of 0.83 (frequency) and 0.84 (intensity) for this scale. [5]. To determine the content validity by faculty members (n = 10), the validity of the questionnaire was verified (0.93). The questionnaire was provided to 20 participants to confirm the reliability (internal consistency), and the Cronbach's alpha coefficient was calculated to be 0.89.

Smith's questionnaire is considered as a valid instrument for measuring job satisfaction that has been designed by Kendall et al. (1969) [14] and includes 22 questions about job nature, seven questions about promotional opportunities, 14 questions about supervisor, 11 questions about colleagues, 9 questions about salary and benefits, and 7 questions about conditions of work environment. The scale used for scoring such questions is a five-point likert scale (completely disagree = 1, completely agree = 5). The results have been analyzed using Smith's guide. The reliability of the instrument has been evaluated using Cronbach's alpha and the obtained value were 0.8, 0.89, 0.87, 0.9, 0.9, 0.80 for job nature, supervisor, colleagues, promotions, salary and benefits, and work environment, respectively. In addition, the validity of the job satisfaction questionnaire was 0.93. The original version of the questionnaire has been translated by Zahedi et al. and its reliability and validity has been calculated as 0.94 and 0.96 respectively [15]. To determine the content validity by faculty members (n = 10), the validity of the questionnaire was verified (0.88). The questionnaire was provided to 20 participants to confirm the reliability (internal consistency), and the Cronbach's alpha coefficient was calculated to be 0.90.

### 2.3. Sampling and data collection

The total number of health workers was 339 and of these numbers, 271 were eligible for inclusion. Participants in this study are health care workers admitted to work in rural health centers with Middle school, diploma and associate education, as well as after a two-year training course in health care.

Physicians and nurses are not included in this study.

According to Morgan table, 150 health workers were selected from the five health centers using the random number table. After obtaining the permission of the Ethics Committee as well as permits and a letter of introduction from the Faculty of Health and Healthcare Centers, at first. Then, all eligible healthcare workers were entered into the study to collect the data by the first researcher. The inclusion criteria included more than five years of experience, fluency in the Persian language and the signed consent and those who did not answer more than one-third of the questionnaire questions were excluded.

### 2.4. Data analysis

In this study, from SPSS 19 were used to analyze the data. Descriptive statistics was used to describe the demographic characteristics and the mean scores of burnout syndrome and job satisfaction. The Pearson correlation coefficient was used to assess the relationship between the mean scores of burnout syndrome and job satisfaction. Data were analyzed using dependent and independent t-test, one-way ANOVA and Pearson correlation coefficient

### 2.5. Ethics approval

This study was approved by the Ethics Committee of Kerman University of Medical Sciences (No: IR.KMU.REC.1398.382). The researcher gave oral and written information and obtained written informed consent from all participants before the interviews. Participation was voluntary, and the participants had the right to withdraw at any time.

## 3. Results

A total of 150 given questionnaires were returned. According to the results of this study, of these, 65.3% were female, 88% were married, a majority (37%) were aged between 41 and 50 years, more than half (56.7%) had diploma and about (26%) had 5–10 years of experience and most of them (80.7%) in the main villages were inhabited (Table 1).

**Table 1.** The demographic variables of healthcare workers.

Variable	Subscale	N	%
Age (in years)	25–30	17	11.3
	31–40	55	36.7
	41–50	65	43.3
	51–60	13	8.7
Years of experience (years)	5–10	39	26
	11–15	30	20
	16–20	29	19.3
	21–25	33	22
	25–30	19	12.7
Sex	Male	52	34.7
	Female	98	65.3
Degree	Middle school	54	36.0
	Diploma and associate degree	96	64.0
Marital status	Single	5	3.3
	Married	132	88
	Separated and died husband/wife	13	8.7
Place of residence	Main village	121	80.7
	Affiliate village	11	3.7
	Vicinity village	5	3.3
	Local healthcare center	13	8.6
Years of residence in the location	1–10	8	5.3
	11–20	30	20
	21–30	37	24.7
	31–40	34	22.7
	41–50	41	25.4
Residence status	Personal possession	110	73.3
	Rent/lease	11	7.3
	Residential area within local healthcare center	19	12.7
	Living with parents, relatives, or others	10	6.7
Number of affiliate villages	1	73	48.7
	2	71	47.3
	3 and 5	6	4.0
Number of children	0	65	43.2
	1	34	22.7
	2	19	12.7
	3	16	10.7
	> 4	16	10.7

The mean score of overall burnout syndrome was 74.83 ± 32.68 which was at a moderate level. The highest mean score of burnout syndrome was related to personal performance and emotional exhaustion, respectively and the lowest was depersonalizing the character. The mean score of overall job satisfaction was 227.39 ± 52.31 which was at a moderate level. The highest mean score of job satisfaction was related to job satisfaction and supervisor satisfaction, respectively and the lowest was salary and benefits satisfaction (Table 2).

**Table 2.** The mean score on dimensions of burnout syndrome and job satisfaction.

Variable	Domain	Mean	SD
Burnout syndrome	Emotional exhaustion	24	14.43
	Personal performance	42.19	12.47
	Depersonalize the character	8.64	5.78
Overall burnout syndrome		74.83	32.68
Job satisfaction	Job satisfaction	73.24	19.44
	Supervisor satisfaction	51.39	14.26
	Colleague satisfaction	40.50	12.96
	Promotion satisfaction	21	8.31
	Salary and benefits satisfaction	19.29	10.28
	Work conditions satisfaction	22.24	8.93
Overall job satisfaction		227.39	52.31

The results of Pearson's Correlation Coefficient shows a negative significant correlation between the emotional exhaustion and supervisor satisfaction (R = −0.233, P = 0.004), colleague satisfaction (R= −0.168, P = 0.041), and overall job satisfaction (R = −0.221, P = 0.007). This means that with increasing emotional exhaustion, supervisor satisfaction, colleague satisfaction, and overall job satisfaction decrease. Besides the personal performance had a negative significant correlation with job satisfaction (R = −0.170, P = 0.038), and salary and benefits satisfaction (R = −0.178, P = 0.029). This means that with increasing personal performance, job satisfaction, and salary and benefits satisfaction decrease (Table 3).

**Table 3.** Correlation between burnout syndrome and job satisfaction dimensions.

Variable	Job satisfaction	Supervisor satisfaction	Colleague satisfaction	Promotion satisfaction	Salary and benefits satisfaction	Work conditions satisfaction	Overall job satisfaction
Emotional exhaustion	R = −0.151	R = −0.233	R = −0.168	R = −0.114	R = −0.132	R = −0.118	R = −0.221
	P = 0.65	P = 0.004	P = 0.041	P = 0.165	P = 0.108	P = 0.152	P = 0.007
Personal performance	R = −0.170	R = −0.026	R = −0.030	R = −0.151	R = −0.178	R = −0.041	R = −0.144
	P = 0.038	P= 0.756	P = 0.715	P =0.064	P =0.029	P =0.619	P =0.078
Depersonalize the character	R = −0.076	R = −0.107	R = −0.072	R = −0.008	R = −0.157	R = −0.073	R = −0.069
	P = 0.354	P = 0.194	P = 0.381	P = 0.282	P = 0.055	P = 0.372	P = 0.404

Note: R = Pearson Correlation.

According to the results of the ANOVA test, the mean score of the personal performance was significantly different in age groups from a statistical perspective (p < 0.05), with the highest difference of 45.49 ± 9.48 and the lowest difference of 12.46 ± 16.14 in those aged 31–40 years. Tukey's follow-up test showed that there's a significant statistical difference between age groups of 30–40 and 41–50 years in terms of the personal performance (p < 0.05).

The mean score of the emotional exhaustion and personal performance according to the educational degree was significantly different in the studies sample (p < 0.05). Tukey's follow-up test showed that there's a significant difference between the mean score for the emotional exhaustion in different degrees of elementary, middle and high school and those with BA and MA degrees (p < 0.05).

Also, there's a significant difference between the mean score of personal performance in those with elementary school degree and other degrees such as middle and high school and university degrees (p < 0.05). The mean score of personal performance was significantly different based on the years of experience in that job.

In addition, the job satisfaction in colleague dimension was significantly different based on years of experience (p < 0.05). Tukey's follow-up test also showed that there's a significant difference between the score of individual performance with 5–10, 11–15, and 26–30 years of experience with those with 21–25 years of experience (p < 0.05). Also, there was a significant difference between the mean score for the colleague dimension, for those with 5–10, and those with 11–15 years of experience (p < 0.05). The results illustrated that there is not any significant relationship between the score obtained for job burnout and job satisfaction dimensions of the study participants and their residential status (p > 0.05). Also the results of study show any significant difference between single and married individuals, and didn't show a significant relationship between the mean scores for job burnout dimensions and job satisfaction of the study population (p > 0.05). There was a positive significant relationship between emotional exhaustion and job satisfaction (R = 0.182, P = 0.026), and a negative significant relationship between the intensity of personal performance with job satisfaction (R = –0.157, P = 0.032), supervisor (R = –0.226, P = 0.005), salary and benefits (R = –0.235, P = 0.004) and general job satisfaction (R = –0.249, P = 0.184) considering the number of children they have. The results didn't show a significant relationship between the mean score for various dimensions of job burnout and job satisfaction of the study participants considering their workplace (p > 0.05), and a significant relationship between the mean score for promotion of salary and work conditions among male and female healthcare workers (p < 0.05).

## 4. Discussion

Considering the role that healthcare workers play in doing preventive actions in health system and the fact that their job promotion will improve the quality of health services provided in healthcare system, the objective of this study was to investigate their job burnout and job satisfaction and their mutual relationship among healthcare workers.

The result of this study showed that healthcare workers gained an average score on job burnout, average score of emotional exhaustion, low score of personality transformation and personal performance. The results were congruent with the results of the number of studies [16]. From the perspective of researchers, personality factors are relatively stable traits that influence the behavior of health care professionals. Some factors, such as neuroticism, have a strong relationship with nurses' job burnout. Knowing how one's personality can affect the development of this more common phenomenon is a challenge. At the same time, it is an opportunity to optimize and improve human resources within the organization [17]. In discussing the aforementioned result, we can argue that job burnout is basically a prevalent phenomenon among aiding professionals including healthcare workers. In addition, the high burnout of personal performance in comparison with the other two dimensions may be the result of the population's lack of control on the work environment, existing laws and regulations and their inability in changing their clients. That's because the ability to control work events is considered as one of the most significant factors influencing personal performance. What is notable here is the high rate of reduction in personal adequacy in the present study and many studies conducted nationally and internationally [18]. In comparison with similar studies conducted overseas [16], showing that reduction of emotional exhaustion and personal transformation is more than the reduction of personal performance, these factors may be influenced by cultural and occupational conditions as well as lower professional standards and organizational and occupational characteristics.

The results of our study showed that overall job satisfaction is significantly related to emotional exhaustion. In a study students' academic burnout has been associated with stress and distress [19]. However, in a study suggested that all dimensions of job satisfaction are significantly correlated with job stress [20]. These results are different from ours. This difference can be attributed to the difference in the population under study and different research instruments used. They conducted their study among employees of local healthcare centers, while the present study has been conducted among healthcare workers of local healthcare centers. The years of residence in the location does significantly affect job satisfaction of healthcare workers' job satisfaction. Some researchers didn't illustrate any significant relationship between job satisfaction and place of residence [21]. These results are congruent with the results of the present study. Another study found a significant relationship between job satisfaction and place of residence ad they concluded that job satisfaction in urban areas is more than job satisfaction in rural areas [22]. Similar to the results of this study, the another study results showed a significant relationship between job satisfaction and place of residence [23]. It shows that job satisfaction in rural areas is more than urban areas. This difference in results may be because of the difference in the samples under study.

In the present study, job satisfaction was higher in single healthcare workers, but this difference wasn't that significant. Similar to the results of our study, a study found similar results by investigating a sample of nurses [22]. On the contrary, Mousavi et al. (2017) found a significant relationship between job satisfaction and sex in a sample of employees in hospitals [24]. These differences can be explained in the light of different cultures and workplace conditions of the sample under study.

According to the present study, job burnout is higher in married participants compared to the single ones; however, this difference wasn't significant. As congruent to the results of the present study, a number of studies, didn't find any significant relationship between job burnout and marital status [13],[25]. One study reported that burnout among nursing aides is related to organizational, personal, and sociological factors. There is a risk of burnout in younger people and in permanent professionals. General self-efficacy and stress management act as protective factors against the possibility of burnout [26]. According to the results of the present study, among various dimensions of job satisfaction, colleagues dimension gained the highest and salary and benefits gained the lowest score. On the other hand, Daniali et al. (2013) suggested that the highest satisfaction among a sample of healthcare workers from local health centers as for colleagues and the lowest satisfaction was for salary and benefits [20]. These results were compatible with ours and illustrate the existence of economic problems and low salaries and benefits for healthcare workers.

In the present study, the researchers didn't find any significant difference between male and female participants in terms of salary and benefits-related job satisfaction. Daniali et al. showed that overall job satisfaction among male and female participants wasn't significantly different, but female participants had lower job satisfaction regarding the nature of their job [20]. These results are congruent with ours as well. Bjork et al., also pointed out that promotional opportunities significantly influence participants' job satisfaction [27].

The researchers concluded that the most stressful problems for the participants were economic problems (high living expenses, loans, etc.), social problems and occupational problems (such as low salary, high responsibility work, and anxiety about the future of the career) respectively [28]. In our study, there was other factors influence job satisfaction besides job anxiety which requires further investigation. An interventional study showed that the teaching stress immunization program resulted in a reduction of nurses' and healthcare workers' job satisfaction [29].

The present study had a limitation. The self-report method was used to measure the job satisfaction and burnout syndrome.

## 5. Conclusion

The results of this study showed that the level of burnout and job satisfaction in health care workers were moderate so that with increasing burnout, job satisfaction decreases. It's recommended to conduct qualitative investigations in order to recognize the reasons for lack of job satisfaction in different dimensions as well as interventional studies in order to identify the best suitable interventions in order to reduce dissatisfaction and measuring the expenses for increasing the effectiveness of the interventions applied in order to reduce healthcare workers' job dissatisfaction in future studies. The healthcare workers were generally dissatisfied with their job, so that it's necessary to improve various fields of organizational communications, job promotion opportunities, salary and benefits, social acceptability, planning actions for maintaining and supporting the employees, challenging jobs, and paying attention to potential capabilities and experience of the individuals in specifying responsibilities to the people.

## References

[1] Jabari F, Rajaei Pour S, Jafari E (2003). Comparative evaluation of occupational motives in faculty member of Isfahan Universities based on Herzberg theory. Health Inf Manag.

[2] Ranai Eshkini F (2000). Study of directors, managers and head nurses job satisfaction and related factors in Rasht hospitals [dissertation]. Tehran: Tehran Univ Med Sci.

[3] Purgaz A, Hezare Mogadam M, Nastiezaie N (2010). Job satisfaction of nurses working in hospitals in Zahedan. J Urmia Nurs Midwif Facul.

[4] Hannani M, Motalebi Kashani M, Gilasi HR (2011). Evaluating the correlation between burnout syndrome dimensions and demographic characteristics of cashiers in state banks of Kashan. Kaums J (FEYZ).

[5] Maslach C, Jackson SE (1981). The measurement of experienced burnout. J Organ Behav.

[6] Ma CC, Samuels ME, Alexander JW (2003). Factors that influence nurses' job satisfaction. JONA: J Nursi Admin.

[7] Jablkowska K, Borkowska A (2005). Evaluation of the intensity of stress at work and burnout syndrome in the managers. Med Pr.

[8] Tongchaiprasit P, Ariyabuddhiphongs V (2016). Creativity and turnover intention among hotel chefs: The mediating effects of job satisfaction and job stress. Int J Hosp Manag.

[9] Judge TA, Weiss HM, Kammeyer-Mueller JD (2017). Job attitudes, job satisfaction, and job affect: A century of continuity and of change. J Appl Psychol.

[10] Rafii F, Shamsikhani S, Zarei M (2012). Burnout and its Relationship with the Nurses' Characteristics. Iran J Nurs.

[11] Wright KB (2011). A communication competence approach to healthcare worker conflict, job stress, job burnout, and job satisfaction. J Healthc Qual.

[12] Ahmed MA, Alkhamis TM (2009). Simulation optimization for an emergency department healthcare unit in Kuwait. Eur J Oper Res.

[13] Arab M, Kheiri S, Mohammadi G (2012). Job burnout and some of its risk factors on the health workers (Behvarz) in Koohrang County, IR Iran, in 2010. J Shahrekord Univ Med Sci.

[14] Kendall L, Hulin C, Smith P (1969). The Measurement of Satisfaction in Work and Retirement: A Strategy for the Study of Attitudes. J Bus Res.

[15] Zahedi M, Palahang H, Ghafari M (2000). Job satisfaction among health personnel in Chaharmahal & Bakhtiari province, 1998–99. J Shahrekord Univ Med Sci.

[16] Bressi C, Porcellana M, Gambini O (2009). Burnout among psychiatrists in Milan: a multicenter survey. Psychiat Serv.

[17] Pérez-Fuentes MdC, Molero Jurado MdM, Martos Martínez Á (2019). Burnout and engagement: Personality profiles in nursing professionals. J Clin Med.

[18] Ouyang Z, Sang J, Li P (2015). Organizational justice and job insecurity as mediators of the effect of emotional intelligence on job satisfaction: A study from China. Pers Indiv Differ.

[19] Martos A, del Carmen Pérez-Fuentes M, del Mar Molero M (2018). Burnout and engagement in students of health sciences. Eur J Invest Health, Psychol Edu.

[20] Daniali SS (2014). Job satisfaction and job stress among staff of health center.

[21] Mostafanejad M, Janati A, Zarnaq RK (2015). Patient Satisfaction with Food Services in Teaching Hospitals of Tabriz; 2012. Depiction Health.

[22] Bagheri Nesami M (2018). The Association between Mental Health and Job Satisfaction in Nurses Working in Teaching Hospitals Affiliated to Mazandaran University of Medical Sciences in 2016. J Health Res Community.

[23] Behzadfar M, Saebi M (2001). Major effective factors in professional satisfaction of social workers. J Qazvin Univ Med Sci.

[24] Mousavi SZ, Shah Hossini M (2016). The relationship between organizational climate and job satisfaction with mental health of Shiraz university staff in 2014. Pajouhan Sci J.

[25] Najafi M, Forouzbakhsh F (2000). Relationship between staff burnout and mental health in staff of nuclear energy organization, Isfahan. J Shahrekord Univ Med Sci.

[26] Molero Jurado M, Pérez-Fuentes M, Gázquez Linares J (2018). Burnout risk and protection factors in certified nursing aides. Int J Environ Res Public Health.

[27] Bjørk IT, Samdal GB, Hansen BS (2007). Job satisfaction in a Norwegian population of nurses: A questionnaire survey. Int J Nurs Stud.

[28] Rouhafza H, Salehi B, Sadeghi M (2007). Prevalence and severity of emotional stress in Isfahan and Markazi provinces, 2002. J Arak Univ Med Sci.

[29] Mazlom R, Darban F (2012). The effect of stress inoculation program on the quality of life of nurses working in psychiatric wards. Iran J Nurs.

